# Real-Time Algebraic Derivative Estimations Using a Novel Low-Cost Architecture Based on Reconfigurable Logic

**DOI:** 10.3390/s140509349

**Published:** 2014-05-23

**Authors:** Rafael Morales, Fernando Rincón, Julio Dondo Gazzano, Juan Carlos López

**Affiliations:** 1 E.T.S.I. Industriales, Universidad de Castilla-La Mancha, 02071 Albacete, Spain; 2 E.S.I Informática, Universidad de Castilla-La Mancha, 130071 Ciudad Real, Spain; E-Mails: Fernando.Rincon@uclm.es (F.R.); JulioDaniel.Dondo@uclm.es (J.D.G.); JuanCarlos.Lopez@uclm.es (J.C.L.)

**Keywords:** time derivative estimation, high-level synthesis, hardware-in-the-loop

## Abstract

Time derivative estimation of signals plays a very important role in several fields, such as signal processing and control engineering, just to name a few of them. For that purpose, a non-asymptotic algebraic procedure for the approximate estimation of the system states is used in this work. The method is based on results from differential algebra and furnishes some general formulae for the time derivatives of a measurable signal in which two algebraic derivative estimators run simultaneously, but in an overlapping fashion. The algebraic derivative algorithm presented in this paper is computed online and in real-time, offering high robustness properties with regard to corrupting noises, versatility and ease of implementation. Besides, in this work, we introduce a novel architecture to accelerate this algebraic derivative estimator using reconfigurable logic. The core of the algorithm is implemented in an FPGA, improving the speed of the system and achieving real-time performance. Finally, this work proposes a low-cost platform for the integration of hardware in the loop in MATLAB.

## 1. Introduction

The derivative estimation of a measured signal has considerable importance in signal processing, numerical analysis, control engineering or failure diagnostics, among others [1–4].

Owing to the measurement, signals are inevitably corrupted by some additive noises—hardware noise of the equipment, background noises, and so on—and so, filtering is a must.

A number of different approaches have been proposed. A common approach is based on least-squares polynomial fitting or interpolation for off-line applications [5,6]. Another common approach is based on high-gain observers [7–9]. These observers adjust the model by weighting the observer output deviations from the output of the system to be controlled. Other interesting approaches are based on the design of numerical differentiators in the frequency domain under the assumption that an ideal n–*th* order differentiator has a frequency response of magnitude *ω^n^* [10,11].

A novel method on derivative estimation based on extensions of techniques for nonlinear closed-loop parametric estimation was introduced by Fliess and Sira-Ramirez in [12,13]. This method allows computing fast and non-asymptotic derivative estimations of noisy signals, avoiding the requirements of knowing a system model or the statistical properties of the signal to be differentiated. This methodology has been successfully applied for solving different problems in control and signal processing [14–16], including numerical simulations. A disadvantage of this method is that it cannot provide continuously accurate estimates, owing to the fact that the method is based on a truncated Taylor polynomial model for the approximation of the signal to be differentiated, and the approximation loses validity over time. A solution of this problem is presented in [17] to improve the transient behavior exhibited when resetting the estimators. In [18] was implemented the control of a DC motor using a disturbance estimator based on the aforementioned algebraic estimator and an experimental implementation using the MATLAB environment. However, the main drawback when using these platforms is the reduction of the sampling time, which results in the impossibility of performing real-time estimation, due to the complexity of the computational work. In order to address the flexibility of software with the performance of hardware, reconfigurable platforms based on FPGAs have quickly become the main option. FPGA-based systems are faster than a pure software approach in terms of computational power with less power consumption, which makes them very attractive for high-performance computing.

FPGA-based SoCs have become an alternative with a growing demand in the solution of computational systems during the last decade. Systems formed by embedded processors plus reconfigurable logic used to accelerate specific application parts can be found in a single chip. FPGA vendors, such as Xilinx, Altera or Actel, offer hard processors, typically ARM multicores, including an AMBAbus interface, in their logic fabric.

The process needed to accelerate applications using reconfigurable hardware has required so far some expertise in hardware design to obtain maximum benefits from the hardware platform where the design is being implemented. The research community has been working on high-level synthesis tools for more than a decade, in order to close the enormous productivity gap for FPGA design, and recently, releases of several of these tools (such as Catapult-C from Mentor Graphics, Vivado HLS from Xilinx or Synphony from Synopsys) are beginning to show good expectations.

In this work, we introduce a novel architecture to accelerate the algebraic derivative estimator using reconfigurable logic. The core of the algorithm that will be described later will be implemented in an FPGA in order to improve the speed of the system and achieve real-time performance.

The purpose of the hardware implementation is double:

- On the one hand, it is the acceleration of the computations, in order to obtain real-time hardware performance of the whole system.
- On the other hand, the aim is to provide a low-cost hardware platform for a hardware in the loop implementation of a system and make it accessible for users with no previous hardware design experience. Here, the term platform has a broad meaning, in the sense of a heterogeneous solution combining a general purpose node plus a hardware accelerator. However, a concrete implementation has been built to demonstrate the validity of the approach, combining a MATLAB testing framework with a hardware prototyping board to host the accelerated estimator.

Using high-level synthesis (HLS) tools, we developed an algebraic derivative estimator starting from a software description of the algorithm. The MATLAB model does not provide support for real-time, so, in this work, we provide an infrastructure that support the estimation of algebraic time derivatives in real time. This estimator, entirely implemented in hardware, can be used as a plug-in in the MATLAB environment, providing benefits for signal analysis in real time. The plug-in is implemented using the “MATLAB executable” (MEX) file and integrated into the MATLAB system through Gigabit Ethernet, but any communication technology supported by the operating system could be used.

The MATLAB environment allows integration of C language, but in this case, due to the hardware implementation of modules, only a set of synthesizable code is integrated in MATLAB environment. This synthesizable code is described in C, and it is converted into an object file. Once the functionality of the component described in C is validated, it will be synthesized into a register transfer level (RTL) description for simulation. Finally, this synthesized code will be implemented in hardware. In all cases, and in each one of the stages of the validation process, the component is integrated to the modeled system in MATLAB through the MEX file plug-in.

This hardware in the loop testing system introduced in this work is implemented in a low-cost platform formed by a PC and an FPGA. This solution allows one to facilitate the integration of the benefits of acceleration of the algorithm in the hardware plus the versatility of the MATLAB simulation environment for signal processing. In this platform, the FPGA gives support for hard real-time functionality in the simulation and measurement processes that could not be provided by a standard PC.

There are, however, some other alternatives when considering the hardware acceleration of a certain part of a MATLAB computation, as will be described in the related work. Some of them use MATLAB M code as the high-level description algorithm, which will be later synthesized into a hardware implementation. Others take a library-based approach, where the accelerator makes use of a set of highly parameterizable components and vendor-specific hardware translators. The first set of solutions, among which, one can cite the one proposed in this work, allow for more generic algorithms and, hence, provide more opportunities for later reuse. The second set provides a higher degree of tuning of the different components of the system, and results in more optimized accelerators. However, most of them are closed solutions only available for a certain set of development boards, mainly because the proprietary interface between the MATLAB environment and the hardware accelerator generated. Such closeness also affects how the accelerator can be interfaced with other hardware resources, such as, for example, data coming from a sensor or a third party interface.

On the other side, the combination of C to hardware synthesis and the automatic generation of the proper adapters, later described in the Design Flow section, can be easily extended to different types of implementations, such as a fully integrated Hw-Swsolution running on a reconfigurable system-on-chip.

The use of a C-based HLS flow has then certain advantages:

- C, the most common system-level language, and there are many algorithms already available, increasing reuse opportunities.
- The HLS synthesis process provides a higher degree of control of the synthesized hardware interface, thus allowing for the interaction with other hardware components available in the system.
- While it may not provide the optimum hardware implementation, it can be mapped into a large set of hardware platforms

The main contributions of this work can be summarized as follows:

- First, it presents an algorithm for algebraic derivative estimation totally implemented in hardware from a pure C description.
- Next, the algorithm is implemented using double precision floating point arithmetic.
- Finally, it proposes a low-cost platform for the integration of hardware in the loop in MATLAB.

## 2. Algebraic Time Derivative Calculations

In an observable system, the state estimation problem is intimately related to the problem of computing the successive time derivatives of the output and input signals in a sufficiently large number (see [19]). A non-asymptotic algebraic procedure for the approximate estimation of the system states is briefly presented in this section. The method is based on results from differential algebra and furnishes some general formulae for the time derivatives of a measurable signal.

### 2.1. Mathematical Framework

Some steps of the derivation presented in [20,21] are now recalled for the sake of simplicity. Let us consider an arbitrary, arbitrary smooth time signal, *y*(*t*), $y:{\mathbb{R}}_{0}^{+}\to \mathbb{R}$. The signal, *y*(*t*), can be approximated by its truncated Taylor series expansion about an initial time *t* = *t_r_* as:

(1) $$y\left(t\right)\approx \tilde{y}\left(t\right)=\sum_{i=1}^{N}\frac{y^{\left(i-1\right)}\left(t_{r}\right)}{(i-1)!}{\left(t-t_{r}\right)}^{\left(i-1\right)}\cdot 1\left(t-t_{r}\right)$$

where 1(*t* − *t_r_*) is the Heaviside unit step function and *N* denotes the order truncation that determines the accuracy of the signal approximation. It is possible to differentiate *ỹ*(*t*) at least *N*-times with respect to time, so as to obtain an expression that is identical to zero. In the operator domain (*Ỹ*(*s*) = 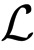
[*ỹ*(*t*)]), this reads as:

(2) $$\left(s^{N}\tilde{Y}\left(s\right)-\sum_{i=1}^{N}s^{N-1}y^{\left(i-1\right)}\left(t_{r}\right)\right)\cdot e^{-st_{r}}=0$$

where the expression, *e*^−*st_r_*^, originates from the time shift to *t* = *t_r_*. Taking into account that *e*^−*st_r_*^ ≠ 0, the following result is obtained:

(3) $$s^{N}\tilde{Y}(s)-\sum_{i=1}^{N}s^{N-i}y^{\left(i-1\right)}\left(t_{r}\right)=0$$

The initial conditions, denoted as *y*^(^*^i^* ^− 1)^(*t_r_*), are eliminated after differentiating *N* times with respect to the complex operator *s*. One obtains:

(4) $$\frac{d^{N}}{ds^{N}}\left(s^{N}\tilde{Y}(s)\right)=0$$

From the previous expression, it is found that the *N*-th time derivative of *ỹ*(*t*) can be expressed in terms of lower order derivatives. Pre-multiplication of *s*^−^*^ν^* gives a recursive system for determining all required time derivatives of *ỹ*(*t*), *i.e.*, the expressions given by:

(5) $$s^{-\nu }\frac{d^{N}}{ds^{N}}\left(s^{N}\tilde{Y}(s)\right)=0,\nu =0,1,\ldots ,N-1$$

contain, respectively, implicit information on the first, second, …, *ν*-th derivatives of *y*(*t*) in an approximate manner. According to [20,21], the following result is obtained,

(6) $$\begin{array}{l} {\tilde{y}}^{\left(i\right)}(t)=\frac{1}{{\left(t-t_{r}\right)}^{i}}\frac{(N+i-1)!}{(N-i-1)!i!}y(t)+\\ +\overset{i}{\underset{j=1}{\text{∑}}}\left(\begin{array}{c} N+i-j-1\\ i-j \end{array}\right)\frac{(N-j-1)!}{(N-i-1)!}\frac{z_{j}\left(N,t\right)}{{\left(t-t_{r}\right)}^{N+i-j}},1,\ldots ,\nu \end{array}$$

where the filter states *z_j_*(*N, t*), *j* = 1,…, *N* − 1, obey:

(7) $$\begin{array}{ll} {\dot{z}}_{j}\left(N,t\right) & ={\left(\begin{array}{c} N\\ j+1 \end{array}\right)}^{2}(j+1)!{\left(-1\right)}^{j}{\left(t-t_{r}\right)}^{N-j-1}y\left(t\right)+z_{j+1}\left(N,t\right),j=1,\ldots ,N-2\\ {\dot{z}}_{N-1}\left(N,t\right) & =N!{\left(-1\right)}^{N-1}y(t) \end{array}$$

which is an (*N* − 1)-th order time-varying, linear filter with homogeneous initial conditions *z_j_*(*N, t_r_*) = 0 for *j* = 1,… *N* − 1. Note that at time *t* = *t_r_*, the above formulae yields an indetermination. In fact, the finite precision of the numerical processors signifies that the computation will not be appropriately defined in a small interval of time of the form: [*t_r_, t_r_* + *ϵ*). The formulae for $\dot{\tilde{y}}$, $\ddot{\tilde{y}}$, *etc.*, are therefore valid for *t* ≥ *t_r_* + *ϵ* > 0. During the interval of time [*t_r_, t_r_* + *ϵ*), we may replace their values with arbitrary constant values or with appropriate function approximations (see [20,21] for details). The issue of how and when to update, or re-initialize, the computations is examined next.

### 2.2. Calculations Resetting

The validity of the formulae for the calculation of the time derivatives, *y*^(^*^i^*^)^(*t*), in the open time interval [*t_r_* + *ϵ, t*) is limited in the time horizon owing to the approximate nature of the truncated Taylor series expansion adopted and owing to the unstable nature of the filter states (7). For this reason, it becomes necessary to reset the computations at a particular finite time, *T*. In this work, an intuitive reset policy is used at equidistant time intervals. This strategy consists of choosing time intervals whose length, T, may be arbitrarily fixed at the outset. The validity of the formulae in each interval of the form [*t_r_* + *ϵ, t_r_* + *T*) is thus assumed. Clearly, *T* ≫ *ϵ*. The determination of *T* may require some additional off-line trial and error runs. This strategy is obviously highly dependent on the encoding system and requires some experience of engineering judgment.

### 2.3. Overlapping Derivative Estimators Technique

From Equation (6), it is clear that, for numerical reasons, a small interval of time, *ϵ*, has to elapse before the results of the estimators become accurate (note the singularity at *t* = *t_r_*). Moreover, depending on the amount of noise, *ξ*(*t*), associated with the measured signal, *y*(*t*), a certain period of integration time is also needed for the filters to attenuate the noise effects. Furthermore, the Taylor polynomial approximation signifies that the approximation of the signal, *Ỹ*(*t*), looses validity when the time difference, *t* − *t_r_*, becomes significantly large. A periodic resetting of the filter states to zero may be necessary at some instant, implying a new *ϵ*-period of integration time before we again achieve accurate estimation results. The following estimation policy is introduced based on [17]: two algebraic derivative estimators run simultaneously, but in an overlapping fashion, so as to obtain valid results at all times except for the very first *ϵ*-interval. The re-initialization of each identifier is separated by a time interval of duration *T*/2. This can be performed by defining two time lines, *t*_1_ and *t*_2_, which are defined as follows:

(8) $$\begin{array}{ll} t_{1} & =t\mathrm{mod}T\\ t_{2} & =t-T/2\mathrm{mod}T \end{array}$$

where the time line for the first identifier is defined as *t*_1_ and, similarly, *t*_2_ for the second identifier. The first identifier is re-initialized when *t*_1_ = 0 and the second identifier when *t*_2_ = 0. The proposed technique resets one of the estimators while the original remains active and *vice versa* (*i.e.*, it resets the previously active one while the second estimator is still valid and non-saturated). This allows the avoidance of discontinuities in the derivative calculation process.

**Figure 1.**
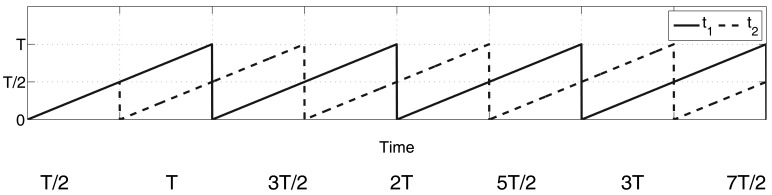
Time setting for each identifier.

This policy is called a switched overlapping estimator technique. Figure 1 depicts the time lines for both identifiers.

### 2.4. Overlapping Derivative Estimator Used in this Research

In our application, only the measured signal, *y*(*t*), is used for the synthesis of the algebraic derivative estimator. The time derivatives of the measured signal, *y*(*t*), are generated by a truncated Taylor series expansion to a seventh order, of *y*(*t*) around the re-initialization time, *t_r_*:

(9) $$\tilde{y}(t)=\sum_{i=1}^{7}\frac{y_{m}^{\left(i-1\right)}(t_{r})}{(i-1)!}{\left(t-t_{r}\right)}^{i-1}$$

which, in the frequency domain, leads to the identity:

(10) $$\frac{d^{7}}{ds^{7}}\left[s^{7}\tilde{Y}(s)\right]=0$$

Based on the previous developments, the derivative calculations in terms of a time varying linear filter are written (according to Equation (7)) as follows:

(11) $${\dot{y}}_{e}(t)=\{\begin{array}{l} \text{arbitrary constant}\;\text{for}\;t\epsilon [t_{r},t_{r}+\epsilon )\\ \frac{1}{{\left(t-t_{r}\right)}^{7}}[42{\left(t-t_{r}\right)}^{6}y\left(t\right)+z_{1}\left(t\right)]\;\text{for}\;t>t_{r}+\epsilon \end{array}$$

(12) $${\ddot{y}}_{e}(t)=\{\begin{array}{l} \text{arbitrary constant}\;\text{for}\;t\epsilon [t_{r},t_{r}+\epsilon )\\ \frac{1}{{\left(t-t_{r}\right)}^{8}}[840{\left(t-t_{r}\right)}^{6}y(t)+35z_{1}(t)+\left(t-t_{r}\right)z_{2}\left(t\right)]\;\text{for}\;t>t_{r}+\epsilon \end{array}$$

(13) $${\dddot{y}}_{e}(t)=\{\begin{array}{l} \text{arbitrary constant}\;\text{for}\;t\epsilon [t_{r},t_{r}+\epsilon )\\ \frac{1}{{\left(t-t_{r}\right)}^{9}}[10080{\left(t-t_{r}\right)}^{6}y\left(t\right)+560z_{1}(t)+28(t-t_{r})z_{2}(t)+{\left(t-t_{r}\right)}^{2}z_{3}(t)]\;\text{for}\;t\geq t_{r}+\epsilon \end{array}$$

where:

(14) $$\begin{array}{ll} {\dot{z}}_{1}(t) & =-882{\left(t-t_{r}\right)}^{5}y(t)+z_{2}(t)\\ {\dot{z}}_{2}(t) & =7350{\left(t-t_{r}\right)}^{4}y(t)+z_{3}(t)\\ {\dot{z}}_{3}(t) & =-29400{\left(t-t_{r}\right)}^{3}y(t)+z_{4}(t)\\ {\dot{z}}_{4}(t) & =52920{\left(t-t_{r}\right)}^{2}y(t)+z_{5}(t)\\ {\dot{z}}_{5}(t) & =-35280(t-t_{r})y(t)+z_{6}(t)\\ {\dot{z}}_{6}(t) & =5040y(t) \end{array}$$

The above formulas for the estimation of the time derivative variables, *ẏ*, *ÿ* and *y⃛*, are valid after a small time interval of duration *ϵ* has elapsed from the instant *t* = *t_r_, i.e.*, during the interval [*t_r_* + *ϵ, t*). A new resetting must be carried out when the validity of the approximation becomes questionable. Assuming that *ϵ* ≪ *t_r_*, the use of the overlapping derivative estimation technique makes it possible to re-initialize without singularities and substantially improves the accuracy of the estimation of the derivatives of the signal. This is carried out by using two identifiers, in a resetting mode configuration, such that the re-initialization of each identifier is separated in a time interval of *T*/2 s (see Equation (8)). Figure 1 illustrates the settings of each of the two time lines. It is necessary to denote the first, second and third derivative estimations of the measured signal, *y*(*t*), as *ẏ_e_, ÿ_e_* and *y⃛_e_*, respectively. The estimations of the first, second and third time derivative signals based on the two identifiers scheme are therefore given by:

(15) $$\begin{array}{ll} {\dot{y}}_{e}(t) & =\{\begin{array}{l} {\dot{y}}_{2e}\left(t\right)\;\text{for}\;(0\leq t\mathrm{mod}T<T/2)\\ {\dot{y}}_{1e}(t)\;\text{for}\;(T/2\leq t\mathrm{mod}T<T) \end{array}\\ {\ddot{y}}_{e}(t) & =\{\begin{array}{l} {\ddot{y}}_{2e}(t)\;\text{for}\;(0\leq t\mathrm{mod}T<T/2)\\ {\ddot{y}}_{1e}(t)\;\text{for}\;(T/2\leq t\mathrm{mod}T<T) \end{array}\\ {\dddot{y}}_{e}(t) & =\{\begin{array}{l} {\dddot{y}}_{2e}(t)\;\text{for}\;(0\leq t\mathrm{mod}T<T/2)\\ {\dddot{y}}_{1e}(t)\;\text{for}\;(T/2\leq t\mathrm{mod}T<T) \end{array} \end{array}$$

## 3. Prototyping Platform

The hardware in the Loop approach has proven to be very effective in the development of complex engineering problems, where an initial mathematical model of a part of the system can be gradually refined into a hardware implementation, while at the same time keeping the advantages of a high-level modeling environment.

The estimator presented in this paper matches this kind of problem, and therefore, one of the objectives of this work was to provide a convenient hardware platform, as well as a high-level design-flow, such that no special hardware design skills would be required to complete the design.

The main difference with respect to other proposals lies in the combination of a low-cost FPGA-based prototyping generic platform, combined with a general-purpose PC, and the use of high-level synthesis tools, as opposed to the proprietary library-based solutions mostly used in HIL platforms.

**Figure 2.**
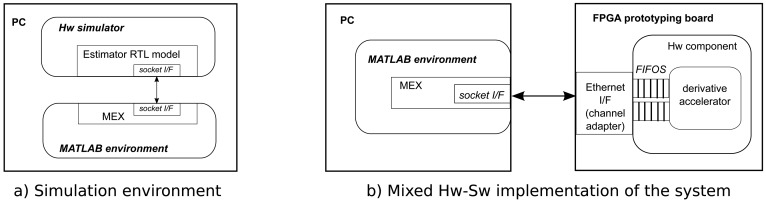
MATLAB Hardware prototyping platform.

The general-purpose PC holds the core of the developing environment, and, hence, runs the MATLAB environment, while the hardware accelerators will be hosted by the FPGA. Additionally, in parallel with the physical interconnection of both computational nodes, the logical link between each part of the system will be carried out through MEX files. Those files are provided as a way to extend MATLAB's functionality, and their primary use is the acceleration of performance-critical routines through the execution of native compiled C/C++ code. Additionally, they provide a way to interface to the modeling environment, which matches the case of the mixed architecture proposed (Figure 2).

As will be later described in the design flow Section 4, MEX files will be used for two different purposes in the prototyping platform:

(1) For the implementation of the C version of the derivative estimator computations.
(2) As an interface to the hardware implementation of the routine functionality. This interface will automatically be generated, depending on the underlying communication technology (PCI, USB, Gb Ethernet) and the signature of the routine (arguments and return values). It can also distinguish between the execution of a hardware simulation running on a third-party tool or the real hardware implementation on the FPGA.

## 4. Design Flow

The implementation of custom hardware accelerators is not a straightforward task, and there are several questions to take into account, such as the functional equivalence of the hardware component with respect to the mathematical version or the interfacing between the hardware and software environment. In order to simplify the complexity of these tasks and to obtain a correct design on the first try, the following subsections describe the suggested design flow.

### 4.1. High-Level Design Model

The initial step would be the definition of the model and the validation environment. This typically implies the writing of one or several MATLAB M files. Here, the only consideration to take into account is that the functionality to be implemented in hardware should have a clear interface and be modeled in a separate file, such that it can be later replaced transparently.

### 4.2. C Code Migration

The main purpose of the MATLAB tool is to provide a powerful and interactive high-level modeling environment, and for that reason, most of the code is run through an interpreter. However, many applications demand higher performance computations, possibly running native compiled C/C++ code. This is done through the use of an extension and an associated compiler, called MEX (MATLAB executable). The use of MEX compiled code may improve performance up to two orders of magnitude when compared to the equivalent M model; however, that is gained at the cost of portability, since the resulting binary files can only be executed on the native architecture for which it was compiled.

It is not strange then to find many applications where native MATLAB code is translated into high-performance C models. Here, the translation is typically done manually, although several contributions have been proposed to automate this tedious and error-prone task.

One alternative for the FPGA-based acceleration of a certain part of the system is the use of the SIMULINK environment and FPGA vendor plugins, such as the DSP library provided by System Generator from Xilinx, so the models can be later compiled into hardware. The main advantage of the solution is the high degree of control of the solution at the expense of extra design effort and less reuse possibilities.

On the other side, the C-based approach taken in this work is more focused on facilitating the use of mixed-mode implementations to designers with no special hardware design skills, than generating the best possible hardware implementation of a function. One of the advantages of the approach is that the resulting model can be used both as a software accelerator or as the source to automatically derive a hardware implementation using high-level C-to-hardware synthesis. In this context, the fact that current high-level synthesis tools provide support for double precision floating point arithmetic, which is the one used by default in MATLAB, thus avoiding the problem of floating-point to fixed-point model conversions, is particularly helpful.

As already described, the interface between C models and the MATLAB run-time is performed through the MEX interface. When the MEX file is only used as a wrapper of a corresponding C function, it can be automatically generated from the signature of that function.

### 4.3. Hardware Synthesis

The current state-of-the-art in synthesis tool technology provides support for a big subset of the C language, excluding those aspects that cannot easily be mapped to a hardware implementation, such as dynamic memory management. Furthermore, the coding style and mapping rules have been simplified, broadening the community of users and not just targeting engineers with a hardware design background. It is only required to understand some basic concepts related to how compilers work.

Most of these tools accept a plain C description that can be shaped into a hardware implementation, through the definition of certain directives, such as the type of protocol, to define for the reception of the arguments, loop manipulation or the identification of blocks that may benefit from a parallel implementation. The whole process of directive definition, synthesis and results analysis takes no more than a few minutes, providing a means to quickly explore the design space, which contrasts the several weeks that the same task would require following classic hardware design flows.

Therefore, the synthesis step in the design flow takes the C code available from the previous stage and generates a register-transfer-level hardware description model, where all operations are described at the clock level. Additionally, a set of directives can be defined to control the performance and cost of the resulting design.

There is a big step from a purely functional model to a hardware description: it is necessary to validate the result of the synthesis before proceeding to the next step. This will require the use of a hardware simulator to run the model and an appropriate test-bench. The solution to this problem is depicted in Figure 2a, where the test-bench is exactly the same MATLAB model used to verify the functional C model, while the hardware component will be simulated using a third party RTL simulator. The interface between them is provided again through the use of a MEX wrapper. In this case, the wrapper implements a message passing mechanism based on a simple socket library. On the simulator side, an equivalent interface has been developed, so messages sent and received through the socket are translated into the activation of hardware signals following a predefined hardware protocol.

All the steps in the process, starting from the C code synthesis and ending in the mixed-mode simulation, are automated through a series of scripts, including the generation of the socket-based MEX file.

### 4.4. Hardware Implementation

The final step in the design flow consists in the real hardware implementation of the model obtained from the C synthesis process. Additionally to the corresponding hardware core, it is also necessary to integrate the logic that will provide an interface between the FPGA-based prototyping board and the PC running MATLAB. There are a number of different solutions completely dependent on the concrete communication technology provided by the board.

The example depicted in Figure 2b would be based on the use of an Ethernet connection, which is the case also described in the Experimental Results section, although a solution based on PCI express, for example, may be more appropriate for applications requiring real-time performance. In any case, both functionality and communication are two separate layers using a well-defined stream-oriented interface, which makes it possible to compose customized solutions from the automatic synthesis/generation of each component in the accelerator architecture.

On possible solution is the case described, where the socket interface in the simulation model is replaced by an Ethernet MAC plus the corresponding FIFObuffers, which can be directly connected to the hardware component generated from the synthesis. This makes the hardware implementation process quite straightforward and completely independent of the concrete functionality implemented by the core. On the PC side, no other modifications than setting the target address for the socket are required. Again, this process is completely automated.

The resulting performance of the mixed-mode implementation will depend on several factors, but the main ones will be the degree of parallelism inherent to the algorithm and the bandwidth and latency of the communication interface between the hardware and the software.

## 5. Experimental Results

### 5.1. Estimator Validation

To assess the validity of the overlapped dynamic estimator, several MATLAB simulations have been performed, where the derivatives of the input signal are previously computed and later checked against the values obtained from the estimator.

**Figure 3.**
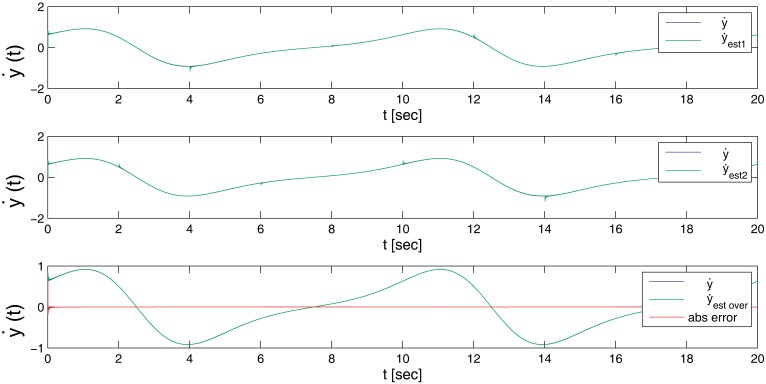
Overlapped estimator for the first derivative.

Figure 3 graphically shows the estimated first derivative with respect to the real one, where the input function was: *e^sin^*^(^*^wt^*^)^ + 0.0001 * *sin*(6000*t* + 2000*t* + 1000*t*). The first and second plots refer to the two estimators running in parallel, while the third one shows the real output of the estimator for the first derivative. The reset time for the estimators has been set to 4 s, and therefore, it is possible to perceive in plots a deviation in the output that quickly converges into the original signal. However, this effect is filtered out in the overlapped estimator.

**Figure 4.**
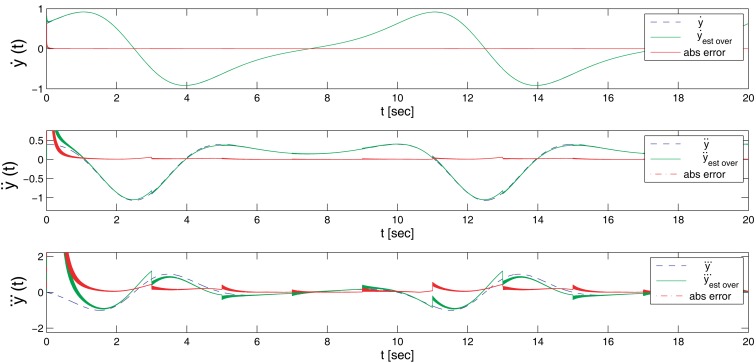
Overlapped estimator for the first, second and third derivatives.

Figure 4 shows the result for the first three derivatives of the signal, compared to the real value, including a representation of the absolute error in each time step. As can be clearly appreciated in the plots, and as described in Table 1, after an initial setup time of nearly 0.4 s, the error quickly drops into values very close to zero. The exact value greatly depends on the time step resolution and to a lower degree on the reset time of the estimator.

Table 1 includes the results for three different cases: the first two using the same time step, but with a different reset time (four and two seconds, respectively), and a third one using a time step of 1 ms. The lower time resolution in the last case improves the simulation speed by one order of magnitude, at the expense of the quality of the estimation.

### 5.2. Hardware Synthesis and Simulation

**Figure 5.**
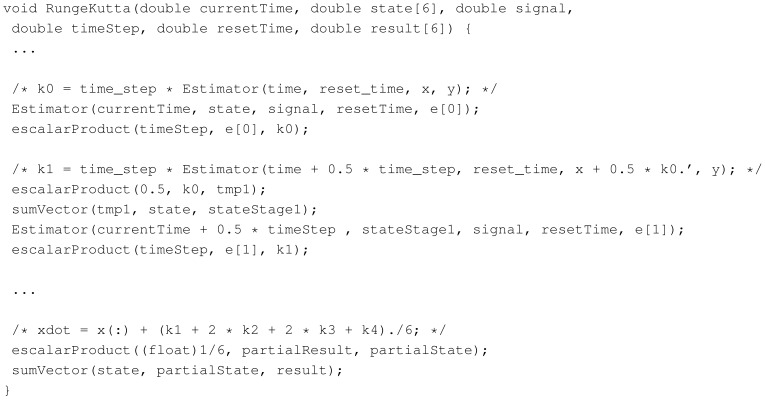
Runge Kutta method C code.

Once the mathematical model has been proven to be correct, the next step for the physical implementation of the estimator was the manual translation into a C-language description. This translation is quite straightforward, but for the fact that C operators do not support matrices types, and therefore, they are replaced by function calls. Figure 5 shows a piece of the C-code that implements the Runge Kutta method used in the estimation, including as comments the original lines in the M-file version. Furthermore, in the code, the estimator call refers to the computations described in Equation (14). Other solutions estimate time derivatives based on algebraic methods using discrete-time FIR filters [22]. This will be one of the topics of our future research.

After the translation of the code, a MEX wrapper for the adaptation of MATLAB types and C arguments was generated, and the whole simulation was run again to prove its correctness.

The C code was synthesized using the default settings of the Vivavo HLS tool. Table 2 shows the results obtained, in both hardware cost and delay latency. Although the design was synthesized as one single component, it is divided into the main three parts, to clarify the contribution of each one to the overall cost. The estimator implements the core of dynamic estimation described in Equation (14) and requires 46 clock cycles (with a 8 ns clock period) to complete its task. The Runge Kutta implements the iterative algorithm for the computation of the estimator, in Equations (11)–(13). Finally, the derivative component includes the overall logic of the overlapped estimator. The overall computation requires 950 clock cycles, since both estimators are executed in parallel, which implies a 7.6-*μ*s iteration time. The computation time to complete the 200 × 10^5^ iterations of the initial example would be of 1.52 s. This time does not include communication overhead that, as will later be analyzed, is communication technology dependent and may become the bottleneck of the accelerator.

The validation of the correctness of the synthesized component was performed according to the procedure described in Section 4.3, using the QuestaSim simulator and replacing the previous MEX wrapper calling the C estimator code with another MEX file that connects MATLAB and the simulator using UNIX sockets.

### 5.3. Hardware Implementation

The hardware platform for the validation of the estimator described in this work combines a general-purpose, 4-GB RAM and i5 processor PC and the Xilinx ML605 FPGA prototyping board. This board provides a big capacity FPGA and several different communication technologies, and therefore, it is a great platform for experimentation. However, it is possible to find really inexpensive boards for no more than a few hundred dollars, such as the Zedboard [23], implementing the necessary reconfigurable fabric, a powerful dual core embedded processor, plus some kind of analog-digital interface support.

Among the different hardware interfaces available in the board, the one finally used was 100 Mb/s Ethernet. Communication between the MATLAB environment and the board has been performed though RAWEthernet messages using a point to point connection. Here, the MEX interface has simply been used to transfer data blocks to and from the board using a RAW socket.

The hardware cost of the system has already been described in Section 2.

To measure the improvement in performance, a full MATLAB version and the mixed Hw-Sw architecture have been run, resulting in around 50 s for the fist case and a bit more than two seconds in the second one. In both cases, the parameters used correspond to those shown in the first case in Table 1, which implies the execution of 2 × 10^5^ iterations of the Runge Kutta-based estimators. From the two seconds, 0.5 where due to the communication overhead introduced by the Ethernet message-passing mechanism, while the remaining 1.5 s purely correspond to the FPGA computation time. This was achieved with a 125-MHZ clock cycle. As an average, the FPGA is able to compute one complete algorithm iteration in 7.6 *μ*s, using floating-point double precision. The communication overhead can be easily reduced using alternative communication mechanisms, such as PCIe bus, for example.

As described in the introduction, one of the purposes of the work was to provide a low-cost solution accessible to non-hardware designers. In that sense, the use of a heterogeneous solution where MATLAB provides the testing environment, while the estimation algorithm is synthesized into a general-purpose prototyping platform, has clear benefits. On the other side, that is not the best use-case for demonstrating the peek performance of an integrated Hw-Sw sensor system. In that case, many systems-on-chip available nowadays would be a better option. However, the use of the developed derivative estimator is not privative of a MATLAB environment. This derivative estimator can also be integrated with those applications running in an embedded processor requiring derivative calculation. The hardware derivative estimator is interfaced through FIFOs, which are later adapted for a concrete communication channel. Those adapters can easily be replaced to fit into any other communication channel technology, such a system bus, as is described in [24]. Therefore, our approach is not based on a tightly-coupled coprocessor, such as in [25] or [26], but is has been oriented to provide a flexible solution, where any low-cost reconfigurable hardware platform can be used as a MATLAB accelerator.

## 6. Related Work

The development of hardware accelerators of specific algorithms is not an easy task, requiring hard work from the designer in order to obtain an optimized component. The optimization process is highly dependent on the target technology and, in general terms, is not easily portable from one technology to another.

Some works found in the literature developed their own compiler in order to automate the hardware generation of accelerated components, such as [27] for signal processing application starting from a MATLAB description. One approach for designing hardware accelerators for numerical computation on reconfigurable platforms starting from a MATLAB description can be found in [28]. In this work the TANORframework is described, enabling the exploration of algorithms and architectural variations to perform efficient hardware implementations.

In [29], the Xilinx System Generated tool attached to the MATLAB/SIMULINK was used with the target purposes of accelerating the functional verification process and of avoiding extra effort to configure the interface to perform the hardware-in-the-loop simulations for dense linear system problems.

The benefits of FPGA make them good candidates to achieve high control performances in industrial control applications. In this kind of system, tools to generate HDLcode, such as the SIMULINK HDL coder, are used to implement the HDL generated components in FPGAs. In [30], an example of using model-based design with MATLAB and an implementation on FPGA of a sensorless controller for current measurement is presented. In [31], a hardware architecture for computing direct kinematics for a robot manipulator is presented using parameterizable floating point units. In this work, the communication with the simulation environment in MATLAB was performed using a RS232 serial interface. This communication scheme only suits low data transference, but it is not suitable for data-intensive computations.

In [32], a system for using a MATLAB target PC in a closed-loop as a controller for a power electronics circuit is illustrated. The key piece in this work is a power interface board, which uses an FPGA to pass data from the power system to MATLAB's controller and, then, from MATLAB back to the converter. HLS tools are a good alternative to create HDL code when using FGPAs, as shown in [33], in which the design and development of control systems for power converters are performed using high-level synthesis. The benefits of HLS tools can be seen in works, such as [34,35], just to name a few of them.

Recent research in the design of time derivative signal estimations based on algebraic methods has been carried out [36–39]. For example, theoretical studies about the achievement of time derivative signal estimations via truncated Jacobi orthogonal series expansion instead of Taylor polynomial series expansion were performed in [38], and some interesting results about the bias error term due to the truncation were obtained. However, not much research has been developed in the experimental design of time derivative signal estimations based on algebraic methods. Works, such as [22,40], can be found in which the Derivative Estimation Toolbox is implemented to determine in real-time time-derivatives of signals, which are validated in a brake test-bench. We have to remark that the previous works presented until the date about time derivative signals based on algebraic methods are mainly numerical simulations and the experimental laboratory platforms performed are based on MATLAB-SIMULINK [41–43]. The development of novel low-cost architectures based on reconfigurable logic, as proposed in this work, will allow the application of these time derivative techniques in a wider range of experimental engineering applications.

## 7. Conclusions and Future Work

This paper implements a novel architecture to accelerate the algebraic derivative estimator using reconfigurable logic. The derivative estimator is based on the use of an overlapping implementation of the algebraic derivative estimation method to compute the time derivatives of the input signals. Two shifted estimators are used, to guarantee that one of them is convergent, while the other estimator is starting to diverge and is thus being properly reset. The outcome of this technique is a considerable acceleration of the convergence of the computation transients that occur just after the estimation resetting. Additionally, the algebraic derivative algorithm presents the following advantages: (i) the algorithm is computed online and in real-time; (ii) high robustness properties with regard to corrupting noises, without the need to know their statistical properties; (iii) versatility and easy implementation; and (iv) it can be robustly applied in a wide range of engineering applications, like signal processing and automatic control.

Complementary to the algorithm, a generic prototyping architecture has been proposed. This architecture might not be the best solution for every case, but as is exposed in the Experimental Results section, it will provide very reasonable results with little effort and cost. The quality of the results will depend both on the concrete algorithm to implement and the communication interface between the hardware and software platforms. In the latter case, there is still room for improvement by the use of Gb Ethernet or PCI express, depending on the delay requirements of the model.

One of the advantages of the HIL approach is the improvement in performance with respect to the native MATLAB models, as is the case of the experiment described in the paper, where the mixed mode solution reduces computation time by one order of magnitude. However, that is not the main purpose of the work, since those speed gains can equally be obtained through C-based accelerators. The real advantage lies in that any kind of hardware can be integrated into the mixed Hw-Sw platform, even for real-time operation. For example, the estimator described in this paper has been tested using data computed from a MATLAB function as the input. However, there is no reason why the input cannot be obtained from the digital hardware in the prototyping board or from a real-time A/D converter. In fact, the ultimate purpose of this work is to provide a mixed Hw-Sw prototyping platform where the estimator can be integrated with real sensors into the same chip, with the purpose of providing enhanced sensors, that provide better quality data.

## Figures and Tables

**Table 1.** Error analysis for the derivatives.

**Derivative Error**	**Median**	**Variance**	**Min**	**Max**
Ts = 0.0001, t_reset = 4 s, error < 0.005 at 0.41 s				

first	−8.7077 × 10^−5^	1.3058 × 10^−6^	−5.0202 × 10^−3^	2.3299 × 10^−3^
second	−9.9813 × 10^−4^	5.3547 × 10^−4^	−2.4609 × 10^−1^	3.4598 × 10^−2^
third	−6.5670 × 10^−3^	1.6780 × 10^−1^	−7.2022	4.1359 × 10^−1^

Ts = 0.0001, t_reset = 2 s, error < 0.005 at 0.41 s				

first	2.8323 × 10^−5^	2.0411 × 10^−6^	−5.0202 × 10^−3^	4.3080 × 10^−3^
second	4.1176 × 10^−4^	1.6769 × 10^−3^	−2.4609 × 10^−1^	1.7306 × 10^−1^
third	3.6795 × 10^−3^	6.5018 × 10^−1^	−7.2029	4.1523

Ts = 0.001, t_reset = 4 s, error < 0.05 at 0.32 s				

first	−4.7759 × 10^−4^	8.01473 × 10^−5^	−5.06375 × 10^−2^	1.3887 × 10^−2^
second	−7.0201 × 10^−3^	6.4461 × 10^−2^	−3.15929	2.7498 × 10^−1^
third	−4.2699 × 10^−2^	4.6275 × 10^1^	−1.1760 × 10^2^	3.3094

**Table 2.** Synthesis results.

**Module**	**Hw Resources**			**Latency**

	**DSP48E**	**FF**	**LUT**	**cycles**
Estimator	39/768	2745/301,440	3046/150,720	46
Runge Kutta	53/768	3942/301,440	4400/150,720	929
Derivative	53/768	4058/301,440	4620/150,720	950
